# Pseudo-Synesthesia through Reading Books with Colored Letters

**DOI:** 10.1371/journal.pone.0039799

**Published:** 2012-06-27

**Authors:** Olympia Colizoli, Jaap M. J. Murre, Romke Rouw

**Affiliations:** Brain and Cognition, Department of Psychology, University of Amsterdam, Amsterdam, The Netherlands; McMaster University, Canada

## Abstract

**Background:**

Synesthesia is a phenomenon where a stimulus produces consistent extraordinary subjective experiences. A relatively common type of synesthesia involves perception of color when viewing letters (e.g. the letter ‘a’ always appears as light blue). In this study, we examine whether traits typically regarded as markers of synesthesia can be acquired by simply reading in color.

**Methodology/Principal Findings:**

Non-synesthetes were given specially prepared colored books to read. A modified Stroop task was administered before and after reading. A perceptual crowding task was administered after reading. Reading one book (>49,000 words) was sufficient to induce effects regarded as behavioral markers for synesthesia. The results of the Stroop tasks indicate that it is possible to learn letter-color associations through reading in color (*F*(1, 14) = 5.85, *p* = .030). Furthermore, Stroop effects correlated with subjective reports about experiencing letters in color (*r*(13) = 0.51, *p* = .05). The frequency of viewing letters is related to the level of association as seen by the difference in the Stroop effect size between upper- and lower-case letters (*t*(14) = 2.79, *p* = .014) and in a subgroup of participants whose Stroop effects increased as they continued to read in color. Readers did not show significant performance advantages on the crowding task compared to controls. Acknowledging the many differences between trainees and synesthetes, results suggest that it may be possible to acquire a subset of synesthetic behavioral traits in adulthood through training.

**Conclusion/Significance:**

To our knowledge, this is the first evidence of acquiring letter-color associations through reading in color. Reading in color appears to be a promising avenue in which we may explore the differences and similarities between synesthetes and non-synesthetes. Additionally, reading in color is a plausible method for a long-term ‘synesthetic’ training program.

## Introduction

Typically described as a ‘crossing of the senses,’ synesthesia is a phenomenon where input from a certain modality induces an experience (i.e. the ‘concurrent’) in another modality or along another dimension of the same modality, which is not normally triggered by the inducer. A relatively common type of synesthesia, grapheme-color, involves perception of color when viewing and/or hearing letters (e.g. the letter ‘a’ always appears as light blue). Synesthesia sheds light on how associations are formed in the brain within and across modalities.

Synesthesia is traditionally considered ‘fixed’ and related to a genetic predisposition [1] and recently to the structure of the brain [2]–[6]. Synesthetic tendencies might be present early in development: manifestations of shape-color associations have been found in two- and three-month-old infants [7]. This might be related to increased connectivity in the brains of young infants, as the associations were not found in 8–month-olds. A study on the prevalence and consistency of synesthesia in children (6–8 years old) found that there is a tendency for the specific synesthetic experiences to ‘solidify’ or become fixed over a period of one year [8]. In this random sample of children, who had recently acquired or were in the course of acquiring linguistic and ordinal inputs, it became apparent that the specific inducer-concurrent pairs of grapheme-color synesthetes develop in close temporal proximity to this cultural learning. These environmental influences do not preclude a genetic factor, but this groundbreaking study highlights the importance of the environmental setting.

Interestingly, in adult synesthetes the majority of synesthetic inducers [9], [10] are culturally transmitted (e.g. letters, numbers, days of the week, months of the year), meaning that the environment (i.e. learning) must play a major role in the development of the particular type of synesthesia. Apparently, while there is a genetic predisposition for synesthesia, the particular inducer-concurrent associations are influenced by the environment [9], [11], [12]. For example, high-frequency graphemes tend to be paired with high-frequency color names [11]. We propose that while there is a genetic predisposition to developing synesthesia, the environment shapes the type of experiences one has [12]. This view is similar to what has been found in language development: the child’s brain may be predisposed to learning a language, however, which particular language the child learns depends on the culture in which he or she grows up [13].

There is at least one documented case of discordant monozygotic male twins, where one twin has grapheme-color synesthesia while the other does not [14]. This raises the question to what level environmental factors can shape synesthetic experiences. One would assume that normally, the genetic predisposition is crucial in developing both the behavioral and the (anatomical) brain differences related to synesthesia. However, there is mounting evidence that not only behavior, but also anatomical brain differences are shaped through learning or other environmental influences [15]–[19]. An interesting question is whether a genetic predisposition is the only route available for acquiring synesthesia. In the current study, we examine whether learning can offer a doorway in creating synesthetic-like experiences and behavior. Furthermore, we examine the degree in which individual differences in learning experiences can shape behavioral properties that have previously been seen in synesthetes.

### Defining Synesthesia

In order to validate of any kind of ‘learned synesthesia’ or ‘pseudo-synesthesia’, then ‘genuine synesthesia’ must be defined. Traditionally, synesthesia has been defined within an individual by the presence of several characteristics (e.g. [2], [20]–[28]): Synesthesia consists of (1) highly specific experiences that are (2) often perceptual in nature, and which are (3) consistent over time (although see [29] for an alternative account). (4) Synesthetic experiences are automatic in the sense that they cannot be turned on or off at will, and (5) have been present since early childhood. (6) Synesthetes seem to have a genetic predisposition to develop synesthesia (it is ‘running in the family’). (7) Synesthetic experiences are not related to any neurological or psychological disease. (8) The neurobiology of synesthesia exhibits distinct patterns in both the function and structure of the brain compared to non-synesthetes.

In order to see the effect of learning synesthetic associations in non-synesthetes, the desired participants would not have (a) the genetic predisposition for developing synesthesia, (b) the functional and structural brain differences found in synesthetes, or (c) a childhood developmental pattern of synesthesia. Training ‘pseudo-synesthesia’ can tell us to what degree these qualities may obstruct shaping synesthetic behavioral properties (characteristics 1–4).

### Training Synesthesia

During lectures or presentations, we often get the question whether synesthesia can be learned. Some types of synesthesia may be beneficial as heuristic tools. For example, grapheme-color synesthetes show some advantages in verbal and/or color memory [30], [31], but see also [32]. In addition, synesthetic experiences are generally reported as pleasant and agreeable, although in some cases synesthetic experiences may interfere with attention and cognition, such as arithmetic in children [33].

Several studies have used training methods to compare trainees and synesthetes (e.g. [28], [34]–[37]). Trainees and synesthetes show differences in brain function [28], [34], [37] and behavior [35], [36]. In general, trainees do not report perceptual experiences that resemble those reported by synesthetes. Perhaps the most well-known example of over-learned number-color associations without synesthetic experience is from the study of Elias et al. [34]. The synesthete and semantic control did not differ behaviorally, but their brain activation did differ on a subset of tasks. Furthermore, only the synesthete reported that visual photisms accompanied the numbers. However, one limitation to this study was the small sample size. Nunn et al. [28] trained 28 non-synesthetes on word-color associations and measured brain activation using functional magnetic resonance imaging (fMRI) while both synesthetes and trainees listened to words. Upon hearing words, synesthetes had additional activation in areas known to be involved in color processing (V4/V8), while controls, even though they were asked to imagine colors for the learned color-word pairs, showed no such activation. Cohen Kadosh et al. [35] trained 15 controls on color-digit associations, in order to test for bidirectional cross-activation between numbers and colors in grapheme-color synesthetes and trainees. It was shown that colors did evoke numerical magnitude in synesthetes, but not in the over-trained control group. In order to investigate the extent to which grapheme-color synesthesia may be learned in non-synesthetes, Meier & Rothen [36] trained four letter-color associations over seven days on the computer. Their results showed that some aspects of synesthesia can be replicated via directed training, such as the (synesthetic) Stroop effect [38]: When asked to indicate the color of a letter, synesthetes respond faster when the letter is in the color of their synesthetic experience (‘congruent’) compared to when the letter is in any other color (‘incongruent’). However, the presence of a synesthetic Stroop effect is not enough to infer the presence of genuine synesthesia, since training an association is enough to produce this effect [39]. Meier & Rothen used a conditioning paradigm, illustrating the apparent differences between trainees and synesthetes. In this paradigm, a startle sound was conditioned to a colored square, corresponding to one of the colors learned during training. A conditioned response, measured with skin conductance, was acquired for the colored square itself, but not for the letter which had been associated to this color during training in non-synesthetes. This is in contrast to synesthetes, who show a conditioned response to both the color associated with a grapheme and the grapheme itself, while the grapheme was never conditioned to the startle sound. Also in contrast to synesthetes, the trainees did not report any *experience* of color while viewing graphemes, which is one of the defining traits of synesthesia. These results suggest that the neurological connections between grapheme and color areas are either not present in trainees, or if present, are not strong enough to induce this synesthetic behavior. Further research is needed to show whether longer training will produce or strengthen these connections.

The perceptual nature of the synesthetic concurrent is perhaps the most striking characteristic of synesthesia, and testing the phenomenological experience objectively has been the goal of most early synesthesia research [25]–[27], [40]. One such task that has been used to test the perceptual nature of grapheme-color synesthesia is the ‘crowding task’. During a perceptual crowding task, a letter in the periphery of the visual field becomes harder to identify when it is ‘crowded’ (i.e. flanked or surrounded) by other letters compared to when it is presented alone [41]. The effect of crowding occurs due to inherent properties of the visual system. When the target letter and flankers are presented in differing colors, detection of the target letter becomes easier [42]. Hubbard et al. [42] showed that synesthetic colors can improve detection performance, albeit not to the extent that real color improves performance. Synesthetes show performance advantages on a perceptual crowding task supposedly because their synesthetic colors help the target letter stand out from the flanker letters. It can, therefore, be attributed to a perceptual effect, in contrast to the Stroop effect, which is considered to be a higher-order cognitive process. If training elicits synesthetic traits, then a difference should be found between letters that the participants were trained to associate with color compared to letters that were never colored. If trainees show this increase in performance on the crowding task, it will be evidence of the perceptual nature of the ‘binding’ of letter to color through exposure to colored letters.

In the present study, we test whether reading books with colored letters can make letter-color associations automatic, perceptual in nature and consistent over time. These traits are objectively tested using a synesthetic Stroop task, a perceptual crowding task and a surprise test of letter-color pair recollection, respectively. In addition, the experiences of the participants were assessed with a questionnaire as a means for quantifying their phenomenological experience in order to see how likely they were to report visual images or ‘photisms.’

Grapheme-color is one of the most common types of synesthesia and the most studied form [43]. Since letters and words are mainly learned through environmental exposure to language (e.g. [44]), we aimed to follow this natural process by presenting written language as a method for synesthesia acquisition. An important change to previous studies is that we wish to study implicit rather than explicit learning. Perceptual learning during reading is mostly implicit because the letter-color associations do not need to be consciously rehearsed even though the participants are aware of the colored letters. The participants are not instructed to remember the color of the letters, but only to read text as they normally would. The goal of this training is to see whether a certain subset of the traits we have defined as synesthetic can be learned.

In order to test the potential of reading in color for learning synesthetic traits, highly motivated participants were recruited to be tested before and after they read specially prepared colored books with four high-frequency letters paired with four-high frequency colors. Each participant had a unique set of letter-color pairs based on their individual preferences. The number of books read varied between participants. Post-session testing was always conducted as soon as possible after the book was finished and included a synesthetic Stroop task and a perceptual crowding task. The results reveal that a Stroop effect for these letter-color pairs is present after reading colored books (>49,000 words). In addition, the Stroop effect correlated with subjective measures on a questionnaire concerning the degree to which participants reported thinking about these letters in color. The results suggest that reading in color appears to be a promising avenue for training grapheme-color synesthesia.

## Results

The significance level for all statistical analyses was set at *alpha* = .05. All t-tests are two-tailed unless otherwise stated. Two participants did not complete the experiment, therefore, all reported results are with *N* = 15. The data reported for the Stroop task are the lower-case letter condition unless otherwise stated. All reaction time data is reported in milliseconds.

### Reading

Participants read an average of 105,660.00 words (*SD* = 69,155.96) and 477,989.87 characters (*SD* = 307,964.34) within two to four weeks. A summary of the reading statistics can be found in **Table 1**. In addition, some participants read Internet pages in color, although we do not have a quantitative means of assessing how much reading was done online. However, only five participants reported using the Internet application, and no one reported using it often.

**Table 1:** Amount of reading and Stroop effects.

Subject	No. Books Read	Word Count	Character Count	Stroop RT (ms)	Stroop %
1	1	38,758	173,522	−26.05	−2.78
2	1	53,727	243,433	5.55	−4.17
3	1	58,363	266,580	12.38	2.78
4	3	181,248	807,463	184.61	22.22
5	1	76,926	356,570	14.59	5.56
6	2	130,653	600,003	58.07	−1.39
7	2	88,372	399,015	180.06	18.06
8	2	127,521	564,030	16.75	0
9	1	76,926	356,570	78.04	0
10	5	282,422	1,272,296	11.69	4.17
11	1	58,363	266,580	92.31	1.39
12	1	76,926	356,570	−21.78	−1.39
13	1	76,926	356,570	86.49	2.78
14	3	208,155	925,153	80.78	11.11
15	1	49,614	225,493	75.3	5.56
Mean	1.73	105,660.00	477,989.87	56.59	4.26
St. Dev.	1.16	69,155.96	307,964.34	64.42	7.54

The character count does not include spaces. Stroop effect data reported here are from the lower-case letter condition after reading. Stroop RT is the difference in reaction times (ms) of incongruent and congruent trials. Stroop % is the difference in accuracy between congruent and incongruent trials. The number of times a participant read each letter is equal to the character count per participant multiplied by the relative letter frequency. Estimated relative letter frequencies are: ‘e’ = 12.07%, ‘t’ = 9.06%, ‘a’ = 8.17%, ‘s’ = 6.33%.

### Stroop Task

**Figure 1:**
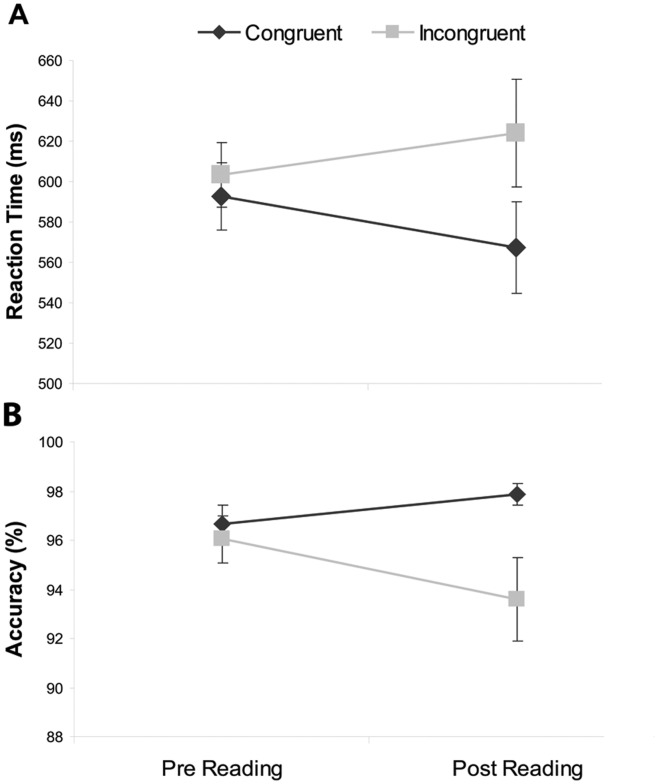
The Stroop Effect. Error bars indicate standard error of the mean (*N* = 15). (A) Reaction times on congruent and incongruent trials, before and after participants had read the colored books. (B) Accuracy on congruent and incongruent trials, before and after participants had read the colored books.

A modified ‘synesthetic Stroop’ [23], [24], [38] version of the Stroop task [45] was given to participants before and after reading. The difference between incongruent and congruent trials is the Stroop effect. A repeated measures ANOVA was carried out for lower-case letters, with testing session and congruency as the levels of interest. As expected, reaction times showed a significant interaction between testing session and congruency (*F*(1, 14) = 5.85, *p* = .030; **Figure 1A**). Participants reacted significantly slower (*t*(14) = 3.402, *p*<.01) to incongruent trials (*M* = 624, *SD* = 103) after reading compared to congruent trials (*M* = 567, *SD* = 87). Participants’ accuracy was significantly better (*t*(14) = 2.19, *p* = .046) on congruent trials (*M* = 97.9, *SD* = 1.73) than incongruent trials (*M* = 93.6, *SD* = 6.74) after reading, although the interaction between testing session and congruency did not quite reach significance (*F*(1, 14) = 3.70, *p* = .066). This is most likely because accuracy was very high in both conditions (**Figure 1B**).

### The Stroop Effect and the Amount of Reading

A positive correlation between word count and Stroop effect size would imply that the number of words read in color directly relates to the strength of the associations between letters and colors. However, no significant correlations were found in reaction times or accuracy when word count was correlated with Stroop effect size. The Stroop effect per participant is given in **Table 1** along with the amount of words read.

In addition to word count, it is possible to test whether reading more increases the Stroop effect size by comparing the data of the participants who were tested again after reading additional books (*N* = 6). For these six participants, we compared their first post-reading Stroop scores to their last post-reading Stroop scores; the difference between the Stroop scores in the first (*M* = 10.78, *SD* = 26.13) and last post-reading session (*M* = 88.66, *SD* = 77.01) was significant for reaction times (*t*(5) = 2.71, *p* = .042); there was an increase in the Stroop effect for all six participants in reaction times (*M* = 77.88, *SD* = 70.4). For accuracy, there was an increase in the Stroop effect for four out of the six participants (*M* = 5.14, *SD* = 12.66), however, the difference between the first and last post-reading sessions was not significant. No significant correlations were found between the difference in the Stroop effect between the first and last post-reading sessions and the amount of words read between these testing sessions.

### The Stroop Effect and Letter Frequency

Lower-case letters appear more often in novels than upper-case letters and are visually dissimilar (with some exceptions, such as ‘s’ and ‘S’). Therefore, we hypothesized that there would be a significant Stroop effect for upper-case letters and that the size of this effect would be significantly smaller than the Stroop effect for lower-case letters. A significant Stroop effect in reaction times was present in upper-case letters after reading (*t*(14) = 3.17, *p*<.01); participants reacted slower on incongruent trials (*M* = 600, *SD* = 79.4) compared to congruent trials (*M* = 575, *SD* = 78.6). Participants performed significantly better (*t*(14) = 2.21, *p* = .044) on congruent trials (*M* = 97.6, *SD* = 2.00) compared to incongruent trials (*M* = 95.1, *SD* = 3.70) for upper-case letters. As expected, the Stroop effect for upper-case letters (*M* = 25.1, *SD* = 30.7) was smaller than the Stroop effect for lower-case letters (*M* = 56.6, *SD* = 64.4), and this difference was significant (*t*(14) = 2.79, *p* = .014). In accuracy, no significant difference in the Stroop effect was found between upper-case (*M* = 2.5, *SD* = 4.4) and lower-case letters (*M* = 4.3, *SD* = 7.5).

### Letter-color Pair Preferences

Before preparing the books, each participant was asked to rate their letter-color pair preferences (16 questions in total). Preferences ranged from 1–5 (low to high). Two participants rated ‘3’ (meaning no preference) for all 16 combinations. **Table 2** shows average letter-color pair preferences. Participants were randomly split into two groups in order to test for an interaction between letter frequency (high versus low) and letter-color pair preference on the Stroop task, but no significant interactions were found in response times or accuracy (F<1). There were no significant differences between the average ratings for preferred and non-preferred letter-color pairs between the two groups. There were no significant differences between the preference ratings of the two groups for any of the four letters.

**Table 2:** Preference for letter-color pairs.

	Mean (Standard Deviation)		
	HF	LF	Total
Group 1	4.14 (.66)	2.5 (.65)	2.5 (1.06)
Group 2	2.56 (.76)	3.94 (.76)	3.25 (1.18)
Total	2.56 (1.09)	3.22 (1.09)	3.3 (1.11)

HF  =  high frequency letters (‘e’, ‘t’), LF  =  low frequency letters (‘a’, ‘s’). Participants rated their preferences on a 5-pt Likert scale for each of the four letters in each of the four colors: red, orange, green, blue. Participants were randomly split in two groups: Group 1 was assigned their preferred letter-color pairs to the HF condition and their non-preferred letter-color pairs to the LF condition. Group 2 was assigned their preferred letter-color pairs to the LF condition and their non-preferred letter-color pairs to the HF condition.

### Crowding Task

Performance accuracy on letters that were colored in the book was compared to a group of baseline letters that were never colored, while all stimuli were presented in black. The reading group (*N* = 15) was compared to a group of controls (*N* = 30) in order to test for inherent differences between letter conditions. There was no significant difference in the average accuracy between groups, ruling out effects due to repeated testing in some participants in the reading group (*N* = 6). There was a trend towards increased accuracy in identification of letters that had been colored in the book compared to the group of baseline letters, indicated by the interaction between letter condition and group (*F*(1, 43) = 2.87, *p* = .097; see **Table 3**).

**Table 3:** Crowding task accuracy.

Mean (Standard Error)			
Group	Baseline Letters	Learned Letters	Difference
Readers (N = 15)	37.87 (2.78)	46.33 (3.76)	8.47 (2.00)
Controls (N = 30)	38.47 (1.62)	42.17 (1.72)	3.7 (1.41)

Baseline letters were ‘D’, ‘F’, ‘O’ and ‘G’. Learned letters were ‘A’, ‘E’, ‘S’, and ‘T’. All stimuli were presented in black.

### Participants’ Experiences

Participants gave an indication of their experience of reading in color by answering 19 questions using a 5-pt Likert scale. The results of the questionnaire are as follows. Q1) The colored text was aesthetically appealing (i.e. ‘pretty’): *M* = 4.13, *SD* = .52. Q2) I read less than the average person: *M* = 2.27, *SD* = 1.22. Q3) I enjoy reading: *M* = 4.6, *SD* = .51. Q4) I tend to read books from the same genre: *M* = 2.46, *SD* = .99. Q5) The colored text was ugly: *M* = 1.53, *SD* = .52. Q6) I read more than an average person: *M* = 3.53, *SD* = 1.19. Q7) The colored text was distracting: *M* = 2.26, *SD* = 1.03. Q8) I was more motivated to read this book compared to a book with normal black text: *M* = 4, *SD* = .65. Q9) The colored text became less distracting over time: *M* = 4.13, *SD* = 1.13. Q10) I enjoyed the content of the book: *M* = 4.6, *SD* = .51. Q11) I tend to read books from a variety of genres: *M* = 4, *SD* = .65. Q12) I was less motivated to read this book compared to a book with normal black text: *M* = 2, *SD* = .76. Q13) The colored text became more distracting over time: *M* = 1.27, *SD* = .46. Q14) I like the color red: *M* = 4.1, *SD* = .1.03. Q15) I like the color blue: *M* = 4, *SD* = .93. Q16) I like the color green: *M* = 3.87, *SD* = .99. Q17) I like the color orange: *M* = 3.6, *SD* = .99. Q18) I am experiencing color when I see certain letters (in addition to the color of the text): *M* = 1.53, *SD* = .91. Q19) I am experiencing color when I think about certain letters: *M* = 2.53, *SD* = 1.36.

In addition, participants were asked to openly answer the following question: Have you noticed any changes in behavior or experience since you started reading the book? There are some examples of preference to specific letter-color pairs that are worth noting. One participant wrote: “I didn’t like orange before reading this text, but now I do! I also feel very attached to the letter ‘t’ having the color orange.” Similarly, another participant wrote: “I am very comfortable with a red ‘E’ (‘e’) and a green ‘A’ (‘a’).” In contrast, a third participant said: “I don’t like the letter ‘f’ (green) so much.” Two participants independently reported the sensation of reading faster. Overall, reading in color was reported as a positive experience. This was reflected in answers to Q8 and Q12 and also by the fact that 40% of our participants chose to read at least one more book and return for additional testing sessions without financial or other types of compensation. However, two of the original 17 participants did not return for the post-testing session and reported to the researcher that they found the books too distracting too read.

**Figure 2:**
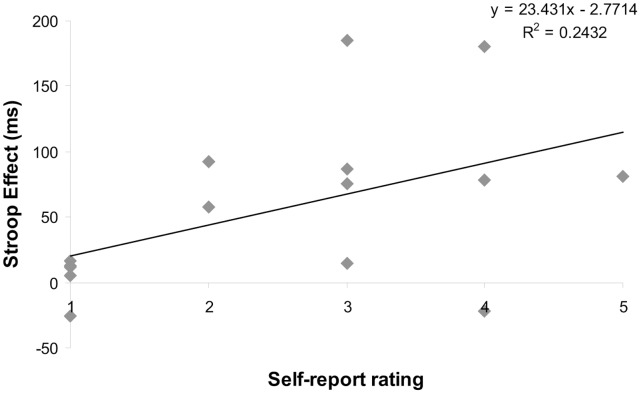
The Stroop effect versus self-report rating of color experience. Participants indicated on a 5-pt Likert scale how much they agreed with the question: “I am experiencing color when thinking about certain letters”. This question is correlated with the Stroop effect: the difference between congruent and incongruent trials during a color naming task.

Subjective experience is the hallmark of synesthesia. Synesthetes often report that the mere thought of a letter is sufficient to experience a synesthetic percept. One of our questions was designed to target imagery-like experiences. It asked participants to indicate how much they agree with the statement: “I am experiencing color when thinking about certain letters” (scale 1–5). There was a positive correlation (*r_s_*(13) = 0.51, *p* = .05) between answers (*M* = 2.53, *SD* = 1.36) and the size of the Stroop effect in reaction times (*M* = 57, *SD* = 64). The results indicated that people who reported being more likely to internally experience the colors of the letters showed greater Stroop effects (**Figure 2**). This question can be contrasted with a similar statement designed to target perceptually-induced experiences: “I am experiencing color when I see certain letters.” Answers to this question did not correlate with any behavioral effects.

### Letter-color Pair Recollection

Participants were never instructed to remember their letter-color pairs. Four to six months after the last testing session, we asked the participants to recall which color each of the four letters had in their books. Participants were given the four letters only. On average, participants (*N* = 14) accurately reported 1.6 out of 4 pairs (*SD* = 1.28). Mean recollection of the four colors was 98% (*SD* = .27).

## Discussion

The goal of this study was to determine whether reading in color could be used to acquire letter-color associations and whether those learned associations are expressed in behavior similar to that found in grapheme-color synesthetes. Specifically, this study tested certain synesthetic traits, such as automaticity, perceptual experiences, and letter-color pair recollection. Fifteen participants read specially prepared colored books and were tested with a modified Stroop task, a perceptual crowding task, and were asked to report about their experiences of reading in color. In addition, we contacted the participants months later to test whether they remembered their letter-color pairs. The results show, for the first time to our knowledge, that letter-color associations can be formed by reading in color. Furthermore, the level of learning these associations is influenced by the frequency of exposure as can be seen by an increase in the size of the Stroop effect for those participants who continued to read, as well as the difference in the size of the Stroop effects between upper- and lower-case letter conditions. The degree to which an individual reports internalizing these letter-color associations was reflected in behavior on the Stroop task. Unlike synesthetes, who are defined by their high consistency of grapheme-color pairs and performance advantage during a perceptual crowding task, readers do not remember their letter-color pairs several months later nor do they show a significant improvement on the crowding task compared to controls.

One must keep in mind that these results alone are not enough to conclude the presence of synesthesia, since over-learned associations produce a Stroop effect. Learned associations might not be conscious or perceptual in nature as is the case in synesthesia. Interestingly, the questionnaire data from participants correlated positively with the size of this Stroop effect in such a way that people who reported being more likely to internally experience the colored letters tend to show a larger Stroop effect. The demand characteristics of the study as well as the question itself may have influenced the participants to think about the letters in color. However, a significant correlation with the reaction time data is evidence to suggest that the self-report ratings of internal experiences are indeed related to learning letter-color associations. Individual differences are immediately apparent in the data. Some individuals who read relatively more did not show as large an effect as other individuals who read relatively less. Furthermore, word count did not correlate with the Stroop effect. We can infer that some individuals are inherently more or less sensitive to forming these associations than others. During the course of this research, we began to wonder if perhaps an individual’s visual mental imagery is a key factor in understanding the differences in sensitivity to forming letter-color associations. We are currently investigating this hypothesis. Individual differences are a major theme in synesthesia research; the term ‘synesthesia’ encompasses many varieties of the phenomenon. Within grapheme-color synesthesia, synesthetes are commonly classified as ‘projectors’ or ‘associators’ depending on the spatial localization of the synesthetic experience [46]. In addition to projector and associator classifications, grapheme-color synesthetes can be categorized into ‘higher’ or ‘lower’ synesthetes depending on what induces the synesthetic experiences (e.g. ‘A’ may or may not induce a different experience than ‘a’) [42], [47]. Even within these categorizations of grapheme-color synesthesia, much variation in the specific qualities of the experiences exists. The best example of this is the fact that the specific colors (and also textures) of the graphemes vary across people. In addition, some individuals experience their grapheme-color synesthesia from visual stimuli only or from auditory stimuli only, while it is also possible that both modalities are inducers, and that the mental images of the graphemes are inducers. Despite the wide range of differences found between synesthetes, there are some general patterns present within larger samples of synesthetes. For example, frequent graphemes tend to induce common colors [11] and correlate with color saturation [48] and luminance [49]. For this reason, we choose four high-frequency graphemes and paired them with four high-frequency colors. We expected that the frequency of viewing letters plays a role in acquiring letter-color associations through reading in color.

In line with our hypotheses about frequency effects, differences were apparent in the data between upper- and lower-case letters. The Stroop effect was present in both upper- and lower-case versions of the same letter. However, the effect was significantly larger for lower-case letters, which are more common in novels, suggesting that the frequency of exposure plays a role. An additional explanation, which is not necessarily mutually exclusive, is that the letter-color association for the lower-case version of a letter is transferred via its semantic relationship to the upper-case version of the same letter. We could not test this hypothesis directly in the current study. In addition, we hypothesize that differences in learning color associations for certain letters may be explained by the differences in letter-position frequency of those letters. Upper-case letters are typically found at the beginning of words. In contrast, lower-case letters are found in all the letter positions within words. The frequencies of letter position may interact with the relative letter frequencies of the individual letters. For example, ‘a’ is less frequent than ‘e’, ‘s’, or ‘t’, yet appears most often as the first letter in the English language. There is evidence to suggest that visual attention while reading differs for letter positions [50], [51]. The current data suggest that this interaction may have an effect on the formation of these letter-color associations, but further experiments are necessary in order to fully understand the nature of such an effect.

A crowding task was administered to test whether reading in color could induce low-level visual effects. Although our results did not reach significance, there is evidence to suggest that reading in color may influence performance on the crowding task. Our crowding stimulus was presented for a very short duration (100 ms) and was not easily recognizable, although there are some limitations to this study. One limitation was that we were not able to monitor the participants’ eye movements in order to exclude trials where a saccade to the stimuli was made. Another limitation may be that the letters were presented in their upper-case forms; an upper-case font ensured equal visual angles in the horizontal and vertical axes in order to preserve the effect of crowding and minimize pop-out due to differences in the visual forms of the letters. The Stroop effect was present for upper-case letters, but was significantly less that in the lower-case condition, and this fact could have hindered the results of the crowding task. None of the participants reported any projector-type of experiences; therefore, a trend in the crowding effect is worth pursuing further to determine if such a perceptual effect could be induced by over-learned associations, for example with lower-case letters or with extended training. It is worth noting here that differences between types of synesthetes on the crowding task were also noted in the original research by Hubbard et al. [42]. One synesthetic participant reported identifying the crowded grapheme on the basis of its color, even though he could not identify the grapheme. While another participant reported that she must first identify the grapheme to experience the synesthetic color. This same participant did not show any performance improvements on the perceptual crowding or embedded figures tasks compared to a group of controls. Again, the variation between synesthetes makes the task of defining ‘genuine synesthesia’ difficult, and one must rely on a set of measures in addition to perceptual tasks, including consistency, to determine if someone is synesthetic or not. In our sample, participants did not recall on average half of their four letter-color pairs correctly several months after testing, although they did recall the four colors used almost perfectly. This can immediately be contrasted with the high-level of consistency seen with genuine synesthesia, where an individual will describe for each grapheme in detail the exact same color each time he or she is asked, with many months to years in between.

What is very special about reading in color is the fact that the effects found are a ‘byproduct’ of the reading process. Although learning by reading in color is not implicit in the strict sense of the term (they are indeed aware of the letter-color pairs), the participants were never instructed to remember the letter-color pairs. It can be seen by the results of their letter-color pair recollection test that they do not remember these pairs well several months later. This begs the question: if people are able to ‘implicitly’ learn letter-color associations by reading in color, why do most synesthetic colors seem to be randomly assigned to their inducer? For example, given the prevalence of McDonald’s in today’s society, why do we not all (Americans especially) think of ‘M’ as yellow? A study on children’s books found little evidence to suggest that the synesthetic colors of a sample of Australian synesthetes came directly from exposure to the specific letter- and digit-color pairs contained in these books [10]. One difference between a synesthete and a trained control, which is stressed in the study from Elias et al. [34], is that synesthetes generally do not know where their colors come from, while the control does. However, this is not always true. In one case, specific synesthetic colors were traced back to refrigerator magnets in the individual’s childhood [52]. In another report, two twin boys have similar synesthetic colors as a shared jigsaw toy [53]. A synesthete reported to one of the present authors that her colors were identical to her kindergarten alphabet. She was shocked to realize this years later as an adult when she went back into the classroom of her hometown and saw her colors as the alphabet on the classroom wall. It seems plausible that this happens in more cases than are reported or remembered, considering that grapheme-color synesthesia is present since early-childhood. If synesthetic colors stem directly from the environment, even in a small minority of cases, the phenomenon is crucial for understanding why synesthesia develops in some people and why some people seem to be more sensitive to certain stimuli than others. Furthermore, in order to understand how grapheme-color synesthesia may develop in children, we must take into account that the mechanisms underlying how a child learns to read and write are inherently different from those processes by which literate adults form letter-color associations through reading in color.

Reading in color has much potential for a long-term training program, probably more than directed training with a computer. Overall, the participants found it a pleasant and interesting experience and gladly took the opportunity to continue reading in color when asked. It is still unclear whether reading in color utilizes the same underlying neural mechanisms as the experience of grapheme-color synesthesia. Studies have shown differences in the structure of the brains of synesthetes, including difference in grey-matter density [3]–[6] and white-matter connectivity [2]. Structural differences are found in sensory cortices, parietal cortex, frontal cortex, and hippocampus (for a review, see [12]). We are currently investigating to what extent changes in the structural and functional properties of the brain can be induced by reading in color. The human brain has surprising plasticity even in adulthood as seen by recent research on experience-induced neural plasticity from learning or practicing traits, such as changes in grey matter induced by learning how to juggle [15], [17], [54], studying for a medical school exam [16], meditation [18], and aerobic exercise in the elderly [19]. Recently, it has been shown that learning new color names increases grey-matter volume in color areas V2/3 in left visual cortex [55]. Changes in white matter have also been found due to experience-dependent plasticity [19], [56]. In light of these findings, it seems plausible that training synesthetic associations might induce changes in the structural and functional properties of the brain. It will be interesting to know if these changes will be similar or in line with the neurological differences between synesthetes and controls that have already been documented (e.g. [2], [5], [6]).

This study, for the first time, tested reading in color as a method for acquiring associations between letters and color. Behavioral effects due to reading in color can be induced in adults, in a mostly implicit manner and in some cases synesthesia-like experiences are reported. Although these trainees are *not* synesthetic in the traditional sense, many similarities exist and we conclude that reading in color is a promising method that may be used to explore both the differences and similarities between synesthetes and people who have been trained on cross-modal associations.

## Materials and Methods

### Participants

This experiment was approved by the ethical committee of the Department of Psychology at the University of Amsterdam. All participants were informed that they could terminate their participation at any time and gave written informed consent before participating in the research. All participants participated voluntarily and were recruited based on their motivation and desire to read. We recruited 17 participants to participate in a ‘reading in color’ study. Participants were screened for synesthesia, synesthesia in their immediate family, and dyslexia by interview with the researcher. Two of the participants (both female) did not continue with the research after the first testing session. All results presented above were from the remaining 15 participants (10 female and 5 male, *M* = 28.93 years, *SD* = 4.25 years). After the book had been read, participants were asked if they would like to continue the research by reading an additional book. Nine participants read only one book. Three participants read two books. Two participants read three books. One participant read five books. Nine participants were tested one time after reading. Three participants were tested three times after reading, and three participants were tested twice after reading. All results are based on the last post-reading session unless otherwise stated. Additional testing in a subset of participants (*N* = 6) had no effect on the performance of the crowding task (see **Results**). Five participants took the crowding task before reading in addition to after reading. Participants were fully informed of the purpose of the research at the end of the last testing session. For the crowding task, 30 participants were recruited for the control condition and were financially compensated (21 female and 9 male, *M* = 22.53 years, *SD* = 3.68 years). All participants were screened for synesthesia by interview with the researcher.

We choose to have participants read in English, because more participants would be likely to volunteer around the university. English was the second language of 12 of the participants. However, we felt that this would not significantly affect results considering the high level of English proficiency of all participants. Each of the 15 participants had received some form of higher education (college or university) in English and spoke at least two languages.

### Preference for Letter-color Pairs

Before testing began, participants were given a questionnaire which asked them to indicate their preference for the four letters (‘a’, ‘e’, ‘s’, and ‘t’) in all four of the colors used (red, orange, green, and blue; 16 questions, 5-pt. Likert scale). Preferred letter-color pairs were defined as a score of ‘3’ or higher, while non-preferred letter-color pairs were defined as a score of ‘3’ or lower (a score of ‘3’, i.e. a neutral preference, could be included in either category). In order to tease apart any effects of color preference from letter frequency, participants were randomly assigned to two groups: The first group was given two of their preferred letter-color pairs for the highest frequency letters (‘e’ and ‘t’) and two of their non-preferred letter-color pairs for the lowest frequency letters (‘a’ and ‘s’). The second group was given two of their non-preferred letter-color pairs for the highest frequency letters and two of their preferred letter-color pairs for the lowest frequency letters.

### Reading Materials

All books were read in English and taken from the Project Gutenburg website (www.gutenburg.org). Books were colored using a customized Macro in Microsoft Word. Participants could choose their own books from a list that was constructed based on books that the principle researcher herself had read. This was done in order to converse with the participants about the content of the books to ensure that the participants had in fact read the books. In addition to the books, each participant was given a personalized web applet that colors text on Internet pages. They were asked to color the Internet whenever they would be reading a page for a significant amount of time.

### Stroop Task Materials and Design

This task was modeled after Meier and Rothen [36]. Stimulus material consisted of four different letters (‘a’, ‘e’, ‘s’, and ‘t’), presented in separate blocks for the upper- and lower-case versions. A blank screen was presented for 500 ms, followed by a fixation cross for 1500 ms. One of the four letters was centrally presented in black for 200 ms before it changed into one of four colors (red, orange, green or blue). The colored letter remained on screen until the participant responded. All trials were presented in random order over three blocks, with a total of 144 trials (72 congruent trials, 72 incongruent trials). The letters subtended visual angles from 1.54° to 2.31° (*M* = 2.00°, 56.6 pixels). Before beginning the Stroop task, participants were trained on the keys that correspond to the four colors. Each trial began with a blank screen for 500 ms, followed by a fixation cross for 1500 ms. After fixation, a colored square in one of four colors appeared on screen until the participant made a response. After a response had been made, the feedback screen appeared for 1000 ms.

### Stroop Task Procedure

Before beginning the Stroop task, participants were trained on the color-button responses in order to accurately record reaction times during the Stroop task. Participant had to learn which of the four buttons corresponded to the four colors used (red, orange, green and blue). They were instructed to be as fast as possible and received feedback on each trial. Participants completed one round of 192 trials and were monitored during the task to see if they were looking at their fingers before responding. If this was the case, they were asked to do another set of training. After training, the Stroop task was administered. For the Stroop task, participants were instructed to identify the color that the letter is presented in as fast and as accurately as possible. A Stroop task for upper-case letters was administered after reading. The order of the upper- and lower-case blocks was counterbalanced across participants. Only correct trials were included in the reaction time analyses.

### Crowding Task Materials and Design

This task was modeled after Hubbard et al. [42]. Stimuli consisted of a target letter (‘A’, ‘E’, ‘S’ and ‘T’; uppercases) surrounded by one of the remaining three letters (12 combinations in total), forming a cross. We included a baseline condition of letters (‘D’, ‘F’, ‘G’, ‘O’; uppercases), on which we would not expect visual pop-out to occur since none of the four letters in that condition were colored in the book. We used an upper-case font in order to ensure equal visual angles in the horizontal and vertical axes which maximizes the effect of crowding and minimizes pop-out due to differences in the visual forms of the letters. Each condition was presented in a single block of 72 trials. Each letter pair was presented 6 times, and equally often on both the left and right of fixation (9.13°, 258.7 pixels). The 12 letter pairs and side of fixation were presented randomly across the 72 trials. All stimuli were black on a white background. The four flanking letters were always the same as each other, but never the same as the target letter. A blank screen was presented for 1500 ms, followed by a fixation cross with a jittered duration to prevent anticipatory eye-movements (random integer between 500 and 1500 ms). After this fixation period, the stimuli were presented randomly for 100 ms on either the left or right side of fixation. After the crowding stimuli, a blank screen was presented for 250 ms before a 4-alternative-forced-choice (4-AFC) response screen appeared. This 4-AFC screen went away upon response or 3000 ms. The vertical size of the letters in the crowding task subtended a visual angle of 1.02° (38.5 pixels), and the average center-to-center spacing of the letters subtended a visual angle of 1.53° (57.7 pixels).

### Crowding Task Procedure

Participants were instructed to identify the middle letter in the group of letters appearing to either the left or the right of fixation. They were instructed to fixate while the letters appeared, and we used a short onscreen duration (100 ms) to reduce the chances of saccades during the stimulus presentation window. Participants first saw two examples of trials in slow motion. Then they were given 20 practice trials before beginning the task. The conditions ‘learned’ and ‘baseline’ were presented in two separate blocks. The order of these two conditions was counterbalanced between participants, with a short break in between these two blocks.

### Reading Experience Questionnaire

At the end of the last testing session, participants were given a questionnaire designed to target their reading experience. All answers were given on a 5-pt Likert scale, where ‘1’referred to ‘strongly disagree’ and ‘5’ referred to ‘strongly agree.’ The instructions were to indicate the extent to which they agreed with 19 statements: 1) The colored text was aesthetically appealing (i.e. ‘pretty’). 2) I read less than the average person. 3) I enjoy reading. 4) I tend to read books from the same genre. 5) The colored text was ugly. 6) I read more than an average person. 7) The colored text was distracting. 8) I was more motivated to read this book compared to a book with normal black text. 9) The colored text became less distracting over time. 10) I enjoyed the content of the book. 11) I tend to read books from a variety of genres. 12) I was less motivated to read this book compared to a book with normal black text. 13) The colored text became more distracting over time. 14) I like the color red. 15) I like the color blue. 16) I like the color green. 17) I like the color orange. 18) I am experiencing color when I see certain letters (in addition to the color of the text). 19) I am experiencing color when I think about certain letters. In addition, participants were asked to openly answer the following question: Have you noticed any changes in behavior or experience since you started reading the book?

### Letter-color Pair Recollection Test

Participants were asked by surprise to report their four letter-color pairs four to six months after the last testing session. The four letters were given, but not the four colors. They were instructed not to check their colors in their books or online before answering. Correspondence was either via email or telephone.

### General Procedure

Four high-frequency letters (‘a’, ‘e’, ‘s’, and ‘t’, in both their upper- and lower-case forms) were paired with four high-frequency colors (red, orange, green and blue). Each participant had a unique set of letter-color pairs based on their preferences for these pairs. The instructions were to read the book. Participants were tested in at least two sessions, once before reading and once after reading (and again if additional books were read). Details of the books were discussed with the participants during the post-reading sessions in order to make sure that they had read the books. The modified Stroop task for lower-case letters was administered in both pre- and post-reading sessions. The modified Stroop task for upper-case letters and the crowding task were administered to all participants after reading. Five participants took the crowding task before reading in addition to after reading. The order of the tasks was counterbalanced across participants. Participants read one page of their books before beginning the computer tasks during the post-reading sessions only. At the end of the post-reading sessions, participants filled out the reading experience questionnaire. Participants were seated 56 cm in front of computer monitor. All stimuli were presented on a PC with Presentation (version 14; www.neurobs.com) on a 16-inch VGA monitor. The screen resolution was 1024×768 pixels. All responses were recorded with a USB keyboard.
